# Domain Mobility in the ORF2p Complex Revealed by Molecular Dynamics Simulations and Big Data Analysis

**DOI:** 10.3390/ijms26010073

**Published:** 2024-12-25

**Authors:** Anna M. Kulakova, Maria G. Khrenova, Maria I. Zvereva, Igor V. Polyakov

**Affiliations:** 1 Chemistry Department, Lomonosov Moscow State University, 119991 Moscow, Russiazvereva@genebee.msu.ru (M.I.Z.); polyakoviv@gmail.com (I.V.P.); 2 Institute of Biomedical Chemistry, 119121 Moscow, Russia; 3 Bach Institute of Biochemistry, Federal Research Centre “Fundamentals of Biotechnology” of the Russian Academy of Sciences, 119071 Moscow, Russia

**Keywords:** ORF2p protein, conformational sampling, open and closed states, molecular modeling

## Abstract

ORF2p (open reading frame 2 protein) is a multifunctional multidomain enzyme that demonstrates both reverse transcriptase and endonuclease activities and is associated with the pathophysiology of cancer. The 3D structure of the entire seven-domain ORF2p complex was revealed with the recent achievements in structural studies. The different arrangements of the CTD (carboxy-terminal domain) and tower domains were identified as the “closed-ring” and “open-ring” conformations, which differed by the hairpin position of the tower domain, but the structural diversity of these complexes has the potential to be more extensive. To study this, we performed sub-microsecond all-atom molecular dynamics simulations of the entire ORF2p complex with different starting configurations. The obtained molecular dynamic trajectories frames were assigned to several clusters following the dimension reduction to three principal components of the 1275 distances feature matrix. Five and six clusters were obtained for the “open” and “closed” ring models, respectively. While the fingers–palm–thumb core retains its rigid configuration during the MD (molecular dynamics) simulations, all other domains display the complicated dynamic behavior not observed in the experimental structures. The EN (endonuclease) and CTD domains display significant translations and rotations while their internal structures stay rigid. The CTD domain can either form strong contacts with the tower or be far apart from it for both formal “open” and “closed” ring states because the tower hairpin position is not the only determining factor of the protein complex configuration. While only the “thumb up” conformation is observed in all the trajectories, the active site can be obstructed by the movement of the CTD domain. Thus, molecular modeling and machine learning techniques provide valuable insights into the dynamical behavior of the ORF2p complex, which is hard to uncover with experimental methods, given the complexity and size of the object.

## 1. Introduction

The long interspersed nuclear element-1 (LINE-1) belongs to non-long terminal repeat (non-LTR) retrotransposons that use the “copy-and-paste” strategy to replicate themselves [1]. Their activity can promote aberrant transcription, insertional mutagenesis, DNA damage and genome instability [2,3,4]. These processes play key roles in the pathophysiology of cancer [5], autoimmune diseases [6], and aging processes [7,8]. Each person inherits about 100 potentially active LINE-1 copies, a small part of the approximately half a million inactive LINE-1 copies and fragments (17% of human genome) [9,10]. The fully functional LINE-1 encodes three proteins: ORF0p, ORF1p and ORF2p [11]. The last two proteins are involved in retrotransposition. ORF1p has nucleic chaperone activity [12] and ORF2p has endonuclease and reverse transcriptase enzymatic activities [13]. This makes ORF2p a potential therapeutic target.

**Figure 1:**
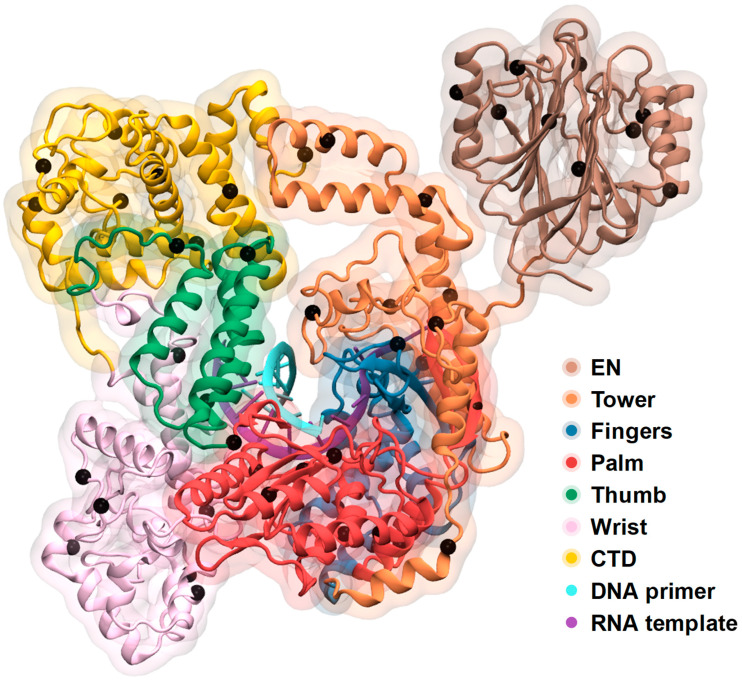
Structure of seven-domain ORF2p complex with DNA-heteroduplex. Black circles depict CA atoms picked as features to machine learning analysis.

The structure of ORF2p consists of five ORF2p core domains, the N-terminus endonuclease domain (EN, 1–238) and the carboxy-terminal domain (CTD, 1061–1275) [14]. The ORF2p core domains include three canonical reverse transcriptase domains: fingers (440–557), palm (558–775), thumb (776–862), and unique domains – tower (239–439) and wrist (863–1061) (Figure 1).

The N-terminus EN domain exhibits endonuclease activity and is the most extensively studied [15,16]. It is responsible for the generation of DNA nicks used by the reverse transcriptase (RT) domains to initiate cDNA synthesis, leading to the production of de novo LINE-1 inserts [2,17]. EN domains demonstrate a tolerance to single and multiple point mutations without a significant impairment of their ability to induce DNA damage [18]. The reverse transcriptase domains use a 3-hydroxyl DNA strand split by the chromosome to create double-stranded DNA [19]. The tower domain has helices and a flexible linker to the EN domain, with amino acids 270–274 appearing to be responsible for the decrease in the cytotoxicity of ORF2p [20]. The CTD domain makes contact with the RNA-template far from the RT active site and helps to unwind the RNA [21]. The reverse transcriptase domains (fingers, palm and thumb) have the same ‘right-hand’ structure as other RTs. The two folded domains, wrist and tower, are absent in other known structures of RT enzymes from viruses or mobile elements [14]. This can cause changes in the balance between “thumb up” and “thumb down” conformations (previously called “open” and “closed” conformations) [22]. These conformations differ in the spatial relationship of the finger and thumb subdomains relative to each other [23]. The “thumb up” conformation is active, that is, the active site is accessible for the RNA-template. In contrast, the “thumb down” conformation is inactive, with the thumb occupying the nucleic acid binding site.

The two domains of the ORF2p, EN and CTD, are stable alone, and are highly flexible in the entire ORF2p, relative to the protein core. This domain mobility has a significant impact on the biological role of the entire protein. The predominant conformation of the ORF2p-RNA complex is the so-called “closed-ring” conformation, with the tower and CTD domains being close to each other. The “open-ring” conformation appears after the participation of ORF2p in retrotransposition [14]. Studying the conformational landscape of a multidomain protein, as well as the transitions from one conformation to another, can bring clarity to the role of conformation in the biological function of ORF2p. The full-atom X-ray structure of the ORF2p core and cryo-EM structure of the seven-domain ORF2p were published recently [14], allowing for the performance of molecular dynamic simulations of the entire complex.

This study aims to explore the conformational landscape of seven-domain ORF2p using molecular dynamics simulations. We obtained a set of trajectories starting from “open” and “closed” states. Due to the system complexity, the feature matrix composed of the interatomic CA–CA distances was suggested. The big data comprising these matrices extracted from trajectory frames was analyzed using the PCA dimensionality reduction technique, followed by gaussian mixture models clustering.

## 2. Results and Discussion

### 2.1. General Analysis of “Open” and “Closed” Model Systems

We considered, separately, the trajectories initiated from the structures prepared from “open-ring” and “closed-ring” conformations (“open” and “closed” model systems, respectively) with respect to the tower domain hairpin positions. A total of 17 and 16 runs were produced for the “closed” and “open” model systems, respectively. Both the apo-form and three complexes were considered for each type of initial state. The complexes were composed of either a protein, DNA and RNA fragments and dTTP nucleoside, or protein, DNA and RNA fragments, or protein and RNA fragments. Protein heavy atoms were extracted from all the trajectories, which resulted in a data set composed of over 61,000 frames in total. The visualization of these frames with the VMD 1.9.3 software shows that the fingers, palm and thumb domains remain in a rather locked conformation while the EN, tower, wrist and CTD domains undergo through several major rearrangements. To accurately define and quantify these conformational changes, we turned to the machine learning techniques and analyzed the entire sets of MD frames, because it was not feasible to produce an adequate description with the naked eye.

We aligned the ORF2p structures obtained in different MD over the fingers–palm–thumb (FPT) core and computed the RMSD values for different domains separately to estimate their flexibility and mobility (Table 1).

The calculated RMSD values demonstrate that while the FPT core complex is rigid, the neighboring and tightly bound tower and wrist domains exhibit pronounced fluctuations. The N-terminal EN domain and the C-terminal CTD domain movements are even much larger due to the translational and rotational degrees of freedom. The domain fluctuations decrease as follows: EN–CTD–tower–wrist–FPT core.

Our main goal is to describe the major conformational changes in the protein complex along trajectories; therefore, the simplest and the most straightforward way to assess the trajectories is through the RMSD metric. TTClust/MDTraj were employed but did not yield meaningful results, which was somewhat expected, given the size of the protein complex and the mobility of terminal domains.

Next, the EnGens workflow (MDTraj/PyEMMA/scikit-learn) [24] was utilized for advanced featurization and clustering; for details, see the Models and Methods section. In short, 1275 features, representing the distances between selected CA atoms of the entire ORF2p protein complex, were utilized, and the dimensionality reduction was conducted with the principal component analysis (PCA), followed by clustering.

### 2.2. Dynamic Behavior of “Open” Model Systems

**Figure 2:**
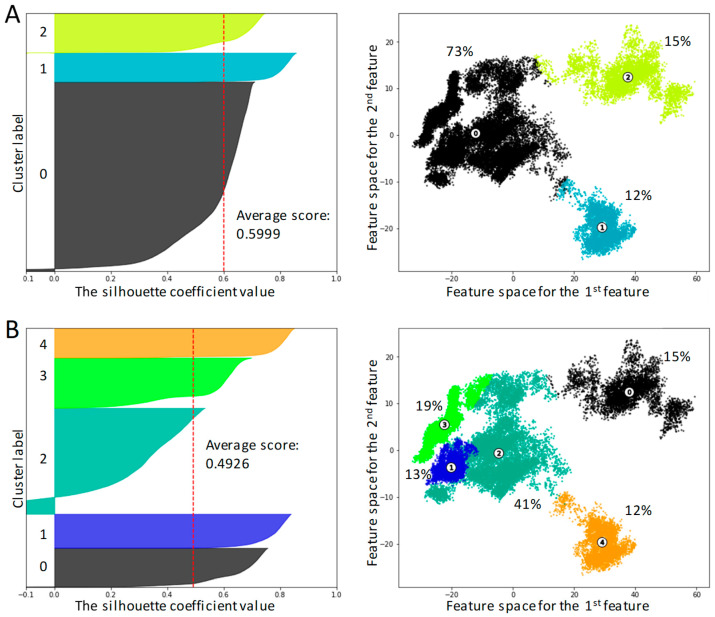
Silhouette and 2D data plots for 3-cluster (panel (**A**)) and 5-cluster (panel (**B**)) partitioning following the PCA feature reduction to 3 components. Cluster weights are shown. Circles with cluster numbers correspond to cluster centers.

The PCA allowed us to identify components that were required to explain the 80% variance threshold; thus, the feature space was reduced to three dimensions and the gaussian mixture models were employed for clustering. The results were assessed with the elbow method and silhouette analysis (Figure 2 and Figure S2).

According to the elbow analysis, five clusters are optimal for data description, while the silhouette score is best for 3-cluster partitioning, with one being predominant (over 70% of conformational space). The second-best silhouette score is obtained for 5-cluster partitioning. Clusters #1 and #2 from the 3-cluster partitioning are the same as clusters #4 and #0 from the 5-cluster partitioning, respectively. The largest cluster (cluster #0) from the 3-cluster partitioning is divided into three clusters, #1, #2 and #3, in the case of 5-cluster partitioning. Cluster #2 is dominant (>40%) in the case of five clusters, while each of the four clusters comprises 10–20% of trajectory frames.

**Figure 3:**
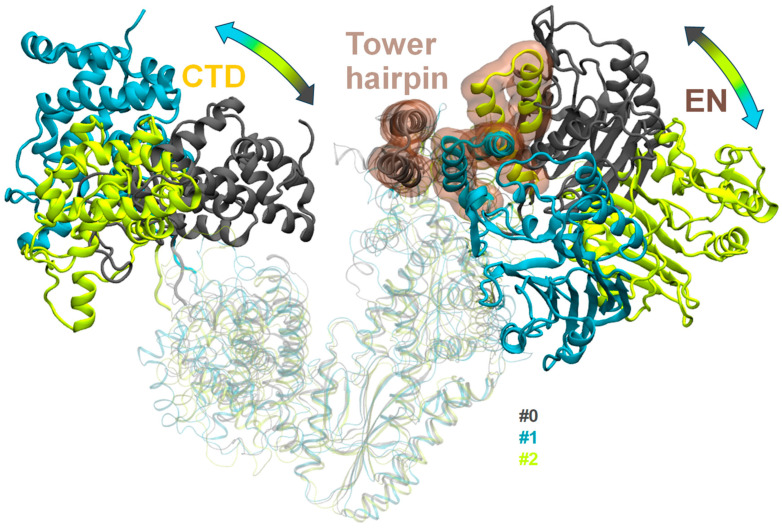
Clusters obtained from three PCA components for “open” model systems in 3-cluster partitioning.

The frames closest to the cluster centers were chosen for further consideration. In the 3-cluster partitioning model, the largest cluster center (cluster #0) structure represents a conformation that is close to the starting point of the MD trajectories, while clusters #1 and #2 are characterized by even more elongated “open” structures than in the beginning of the simulation (Figure 3).

The structures representing the centers of clusters #1 and #2 are characterized by the following: the tower domain moves further away from the thumb and CTD domains compared to in cluster #0 (Figure 3). This correlates with the change in position of the EN domain, but in cluster #1, the EN domain sticks close to the tower domain while in cluster #2, the EN domain moves away from the protein complex, maintaining only the slightest contact with the tower domain. The difference in the position of the CTD domain between the representative structures is significant. The CTD domain forms a strong contact with the tip of the thumb domain in the starting structure and cluster #0, which is no longer the case for both clusters #1 and #2. There are still contacts between the CTD and the loop at the end of the thumb domain in cluster #1 while in cluster #2, the CTD domain moves even further away, breaking all contacts with the thumb. Clearly, the model with three clusters allows one to describe the overall protein complex behavior. Less than one third of the states obtained in MD simulations can be described as elongated “open” conformations (clusters #1 and #2) that are clearly seen from the representative frames. Still, this model fails to describe the diversity of the structures in the dominant cluster #0, which is evidently observed from the visual trajectory inspection.

**Figure 4:**
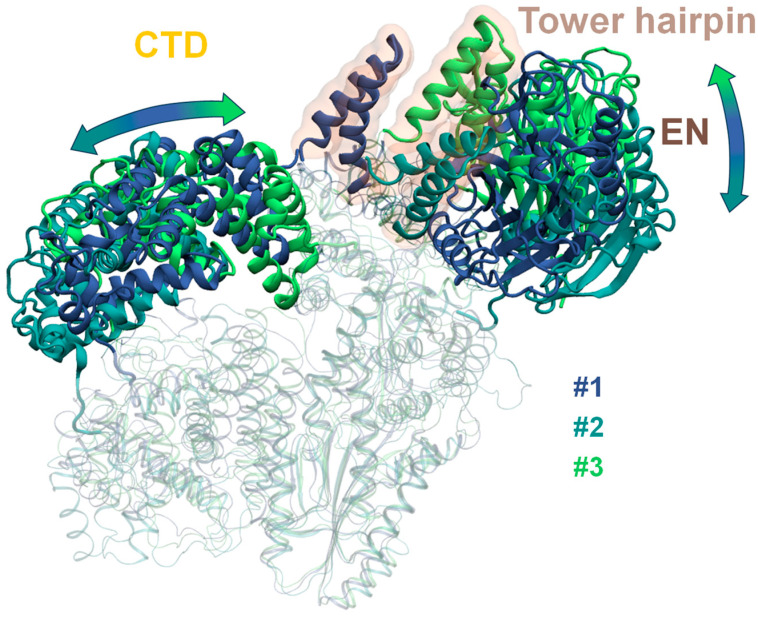
Clusters #1, #2 and #3 obtained from three PCA components for “open” model systems in 5-cluster partitioning.

Therefore, we demonstrate the results of the 5-cluster partitioning, paying special attention to the division of the major cluster #0 from the 3-cluster partitioning into three clusters, #1, #2 and #3. This allows one to obtain a more detailed description of the cluster #0 conformations (Figure 4).

**Figure 5:**
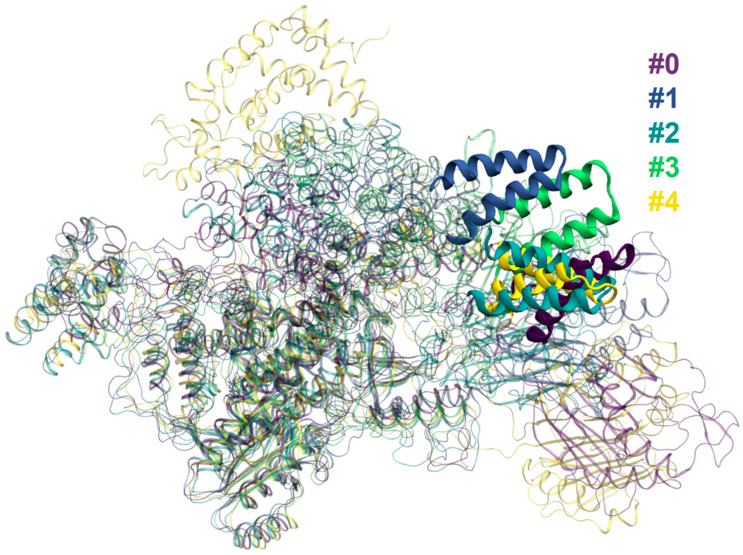
Alignment of representative frames for “open” model systems in 5-cluster partitioning. Conformations of the representative structures of the double α-helix hairpin of the tower domain are highlighted.

The cluster #2 center structure is close to the starting point of trajectories while the main feature of the conformations of the centers of clusters #1 and #3 is the change in position of the CTD domain. In cluster #1 it moves closer to the tower domain forming contacts. The representative structure in cluster #3 demonstrates that the CTD domain tip continues to move towards the ORF2p core and gets between the thumb and tower domain. These conformational adjustments come about with the tower and EN domain changing their positions from the starting point, but in a different way, compared to clusters #0 and #4. The EN domain moves in a direction pointing towards the end of the tower domain while in clusters #1 and #3—to the start. The structure of the cluster center does not give us all of the information on the conformations of the entire cluster as the EN domain position visibly fluctuates during the MD trajectory. The RMSD for the backbone of residues comprising the EN domain confirms this (Table 1). The EN and CTD domains are rigid themselves and the main differences are due to their translations and rotations (Figure 5). Contrarily, the tower domain cannot move much but adopts different conformations (Figure 5).

**Figure 6:**
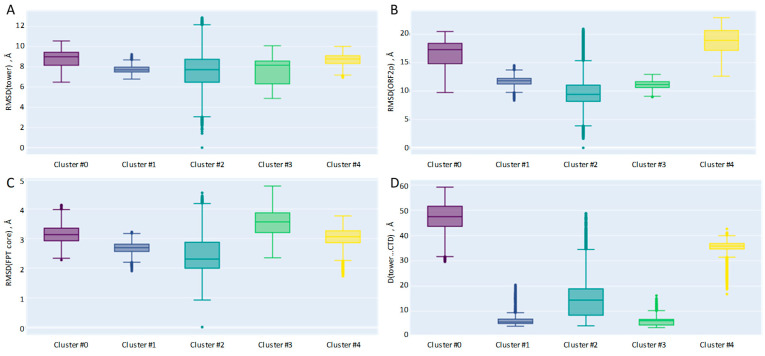
Key geometry features that characterize the differences between clusters. (**A**) RMSD calculated for backbone atoms of tower domain (**B**) RMSD of the entire ORF2p. (**C**) RMSD of the FPT core. (**D**) Closest distance between CA atoms, one being from the tower and the other from the CTD domain. All RMSD calculations were preceded by the alignment of the respective backbones.

The tower domain conformation is stiffest for cluster #1, which could be due to the formation of stabilizing contact with the CTD domain. In cluster #3, the CTD domain moves even deeper into the complex which, apparently, destabilizes the tower domain, leading to more fluctuations. Cluster #2 spans the most frames (>40%), is less well-defined than others (Figure 2B—silhouette analysis plot) and exhibits significant fluctuations in the tower domain (Figure 6A). Unlike the EN and CTD domains, the tower fits tightly with the FPT cluster and cannot undergo significant translations and rotations; instead, the tower domain backbone is way more mobile compared (Table 1, Figure 6A) to the FPT part of the complex, which is very stiff, overall, (Figure 6C) for all the considered trajectory frames.

It should be noted that the cluster #1 and #3 centers can no longer be named as “open-ring” conformations because they restore the contact between the CTD and tower domains in a different way compared to the conventional “closed-ring” conformations, where the tower domain comes closer to the CTD domain and the double alpha-helix hairpin (residues ~314–350) of the tower domain gets tilted toward the CTD domain. The cluster #1 and #3 protein complex conformations get “closed” through the movement of the CTD domain toward the tower, going as far as to enter the pocket formed by the thumb and the tower (closing the active site, see Figure 4, green), while the double alpha-helix hairpin of the tower domain still points away from the CTD domain, not towards it. In the starting frame, the minimum distance between any CA atom of the CTD domain and any CA atom of the tower domain is ~16 Å. In ~38.5% of the “open” start trajectory frames this distance is less than 8 Å (Figure 6D).

The more elongated open conformations (cluster #0) are significantly presented and, in fact, dominant (59%) in the apo-form. The conformations close to the initial starting point (cluster #2) are the most populated for the ternary complex (68%) and least presented for the apo-form (11%). Clusters #1 and #3 are both characterized by the CTD domain movement toward the tower and the FPT core into the active site. Conformations from clusters #1 (58%) and #3 (73%) are dominated in DNA- and RNA-complex landscapes, respectively. Additional details can be found in Table S1.

In Section 2.1 we mentioned that the RMSD metric obtained by aligning the entire protein fails to describe the protein complex configurations. This is due to the fact that the conformations and relative position of multiple domains can change during simulations, deriving the same RMSD values. If we align all of the frames on the FPT core, we can distinguish different states even while using the same RMSD metric, as shown for the different clusters (Figure 6B).

### 2.3. Dynamic Behavior of “Closed” Model Systems

**Figure 7:**
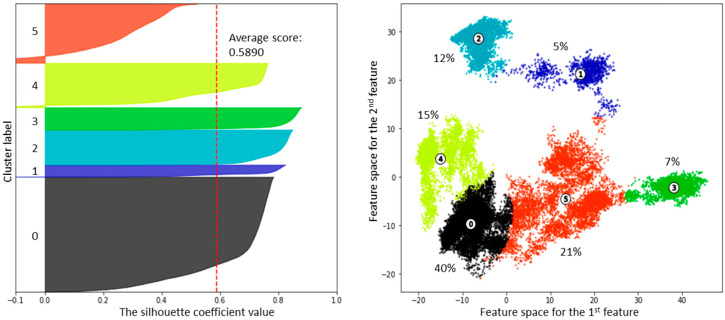
Silhouette and 2D data plots for 6-cluster partitioning following the PCA feature reduction to 3 components. Cluster weights are shown. Circles with cluster numbers correspond to centers of clusters.

The featurization and clustering procedure for the trajectories obtained from the “closed” starting point was carried out with the same procedure as for the “open” starting point. Both elbow and silhouette analyses determined six clusters as an optimal partitioning scheme (Figure 7 and Figure S3, Table S2).

**Figure 8:**
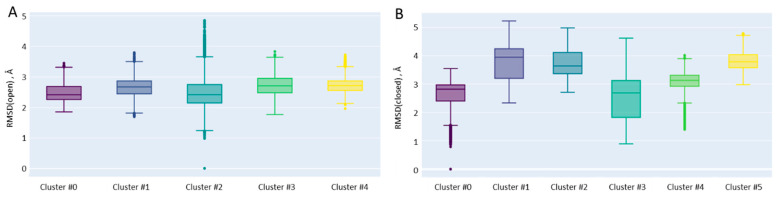
RMSD calculated for the EN domain in MD trajectories derived from “open” (**A**) and “closed” (**B**) model systems. All structures are aligned relative to the FPT core of the initial structure. The overall RMSD value is 2.6 ± 0.4 Å for the trajectory derived from the “open” model and 3.0 ± 0.7 Å for the trajectory derived from the “closed” model.

Cluster #0 dominates in the conformational space and accounts for 40% of the states. Moreover, the starting frame of the MD trajectories is attributed to that cluster. This cluster is the most populated for the RNA complex and least presented in the ternary complex. First, we note that the EN domain (as in the case of “open” model systems) is very motile, and we abandon efforts to describe its position and orientation in different clusters. If we consider the RMSD of the backbone of the EN domain, eliminating the rotations and translations, the “stiffness” is reduced in the trajectory starting from the “closed” model (Figure 8).

**Figure 9:**
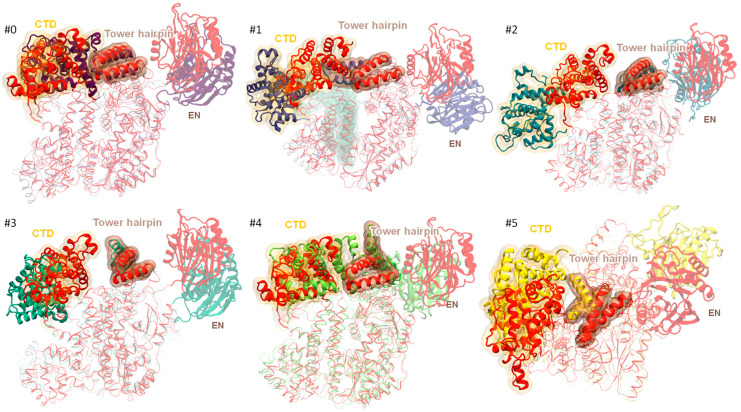
Alignment of the initial structure of the “closed” model (red) and structures representing the centers of six clusters obtained in MD simulations. The CTD and EN domains and tower hairpin are highlighted. Each figure is marked by the cluster number from Figure 7.

The following is the discussion of the CTD domain dynamics and the differences in its states in determined clusters. The main feature of cluster #4’s representative structure is the extensive contact between the tower and CTD domains due to the CTD domain’s movement towards the tower’s hairpin to adopt an even more “closed-ring” and tightly packed structure than the starting one (Figure 9). Cluster #4 is the most populated (64%) in the DNA complex.

Notably, the cluster #3 center structure exhibits a shift in CTD domain position away from the tower hairpin and towards the wrist domain, which adopts a conformation favorable for contact with the CTD. Clearly, the conformations from cluster #3 cannot be classified as “closed-ring” (Figure 8). They are, instead, a variant of the “open” configuration of the protein complex. Cluster #3 is dominant for the ternary complex (67%) and is rarely found in all the other cases. The least populated cluster #1’s representative structure is a peculiar case and is only populated for the apo-complex. The tower domain hairpin locks firmly with the thumb domain and pushes the CTD domain away slightly to allow for such a thumb-tower locked state (Figure 9). The CTD domain moves closer to the fingers domain in the representative structure of cluster #5, leading to a more tightly packed protein complex structure (Figure 9). This is only possible for the apo-complex, and such a configuration accounts for 50% of its population. Cluster #0 demonstrates that the CTD domain is in tight contact with the tower and fingers, forming a locked interface between these three domains. This was already uncovered while analyzing the conformations of the trajectory derived from the “open” model system. The CTD domain can also move far away from the tower and thumb domains to stay close to the end of the wrist, which is the case for the cluster #2 structures (Figure 9).

**Figure 10:**
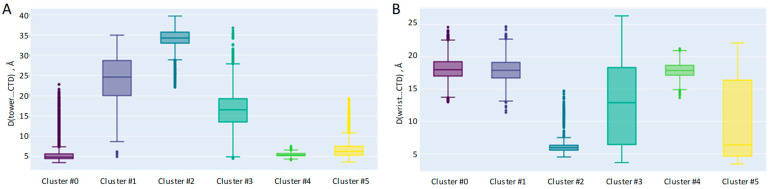
Closest distance between CA atoms, one being from the CTD domain and the other from either the tower (**A**) or wrist (**B**) domains.

We back up the analysis of the representative structures from the cluster centers with the analysis of the distribution of minimum distances between the CTD and other domains; the interdomain distances between the CA atoms are computed (Figure 10).

In ~65% of the MD frames of the “closed” system, the minimum CA–CA distance between the CTD and the tower is less than 8 Å, which confirms a strong contact that we also observed in clusters #0, #4 and #5. Still, that means that starting from the “closed” initial state, we can obtain “open” type conformations that dominate in clusters #1, #2 and #3.

The analysis of the CA–CA minimum distances between the CTD and wrist domains requires adjusting the domain ranges to 1068–1275 and 877–1056, respectively, because we are not interested in the “trivial” contacts. These are the end of wrist–start of the CTD or starting loop of the wrist—these are present in all the frames.

The obtained results (Figure 10B) confirm the visual observation for cluster #2 but add some additional insights—for some points in clusters #3 and #5, the contact between the wrist and CTD can also be tight. Cluster #3 is an “open-ring” conformation, which clearly allows for a significant float in the CTD domain when compared to the locked “closed” configurations, like in cluster #0, #1 and #4. In cluster #5, the CTD domain drifts towards the “fingers”, which also allows it to be closer to the wrist domain.

### 2.4. Dynamic Behavior of the CTD and Tower Domains

Configurations with short distances (less than 8 Å) between the CTD and the tower are observed in clusters #0, #4, and #5 for the “closed” system and clusters #1 and #3 for the “open” system. Domains can adopt different orientations relative to each other and the following is the description of these peculiarities.

**Figure 11:**
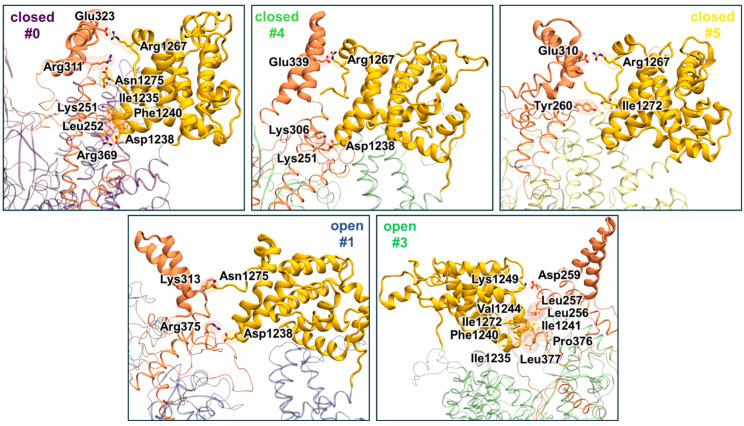
Interactions between CTD (yellow) and tower (orange) domains in clusters with short (less than 8 Å) distances between their CA–CA atoms.

In the “closed” system, the tower hairpin in clusters #0 and #4 is positioned along the CTD domain, but in slightly different ways. In cluster #4, the interaction between the domains is facilitated by salt bridges Lys251-Asp1238, Lys306-Asp1238, and Glu339-Arg1267 (Figure 11). Notably, the hairpin fits snugly to the CTD domain in cluster #0 not only due to salt bridges Arg311-Asn1275, Glu323-Arg1267, Arg369-Asp1238, but also due to hydrophobic interactions between Lys251, Leu252, Ile1235, and Phe1240. In cluster #5 of “closed” system, the CTD interacts with the thumb domain, with only a touch of the tower at the base of the hairpin through the Glu310-Arg1267 salt bridge and a hydrogen bond between Ile1272 and Tyr260.

Cluster #1 of the “open” system has some similarities with cluster #5 of the “closed” conformation. The CTD domain contacts the base of the tower hairpin, but by different contacts: Lys313-Asn1275 and Arg375-Asp1238. In cluster #3 of the “open” system, the loop of the domain tower is positioned away from the CTD domain. The CTD domain primarily interacts with the fingers domain and another fragment of the tower domain. The interaction with the tower domain is maintained by the Asp259-Lys1249 salt bridge and by hydrophobic interactions between Leu256, Leu257, Leu377, Leu380, Pro376, Ile1235, Phe1240, Ile1241, Val1244, Ile1272.

The conformational flexibility of the CTD domain might have a biological role. It is known, from experimental studies, that the CTD domain binds RNA non-specifically with the dissociation constant of the low nanomolar range [25]. From the MD study, we know that the CTD domain forms various transient complexes, characterized by a short distance to the tower domain. These structures presume short distances between the reverse transcriptase active site and the CTD domain. We can speculate that if the RNA binding to the CTD domain occurs in such a conformation, it facilitates the formation of the reactant complex for the following reaction. In other words, the CTD domain attracts the RNA to the active site. According to the clusterization results, in the apo-form of the complex in the “closed-ring” system, closed conformations, with respect to the CTD domain, are observed in 50% of the states. In the “open-ring” system, this value is reduced to 30%. Upon the binding of the RNA (“RNA” model), the “closed-ring” system exists in the “closed” state during the entire simulation. In the “open-ring” system, this value is 72.8%. We can deduce that the “closed-ring” form is more favorable for the reverse transcriptase activity of ORF2p.

Baldwin et al. [14] utilized integrative biology methods, including cryo-EM and subsequent molecular modeling tools, to obtain the entire ORF2p structure and determine the “open-ring” and “closed-ring” states, as well as the extended mobility of the EN and CTD domains. Herein, we extended the understanding of the dynamic behavior of ORF2p and revealed that, apart from the states determined by the tower domain hairpin position, the CTD and tower domains can be close or distant, and that might make biological sense for the RNA binding and its posing to the reverse transcriptase domain. Also, the transitions between “closed-ring” and “open-ring” conformations are observed, indicating that the barrier between these states is low.

## 3. Materials and Methods

The crystal structure (PDB ID: 8C8J) [14] of a ternary ORF2p complex with thymidine triphosphate (dTTP) nucleotide and a RNA_12_–DNA_9_ template–primer heteroduplex was used as the source of initial coordinates of heavy atoms. The unresolved parts of the protein, such as EN, CTD domains, parts of tower and wrist domains, were added from cryo-electron microscopy results available from the ModelArchive repository (https://www.modelarchive.org/doi/10.5452/ma-9wovj (accessed on 22 December 2024)) to obtain “open-ring” and “closed-ring” conformations of the protein, with minimal and maximal distance between the CTD and tower domains. Hydrogen atoms were added using the Reduce program [26] to reproduce the protonation states of amino acids at neutral pH. The following protonation states of side chains were assigned: positively charged Arg and Lys, negatively charged Glu and Asp and neutral His residues. The side chain of the Cys661 residue was in the oxidized S-oxy form.

The DNA complexes of ORF2p in “open-ring” and “closed-ring” conformations were obtained from ternary complexes by removing the dTTP nucleoside, respectively. The RNA complexes of ORF2p contain only RNA-template and protein. We also construct the apo-form of ORF2p in “open-ring” and “closed-ring” conformations. All these eight model systems were solvated in a rectangular water box, with a dimension of 131 × 160 × 146 Å^3^, and neutralized by adding Cl^−^ ions (58—for ternary complex, 62 for DNA complex, 70 for RNA complex and 79 for the apo-form). In total, the ternary complex (“all” in Table S1), DNA complex (“DNA” in Table S1), RNA complex (“RNA” in Table S1) and apo-form (“apo” in Table S1) of ORF2p contained 283,160, 283,110, 282,804 and 282,396 atoms, respectively.

Despite the large system size we utilized all-atom force fields: the CHARMM36 [27,28,29] for the protein, DNA, RNA, dTTP and the magnesium ion; and the TIP3P [30] for water molecules. Classical MD trajectories were simulated with the NAMD 3.0 [31] software package in the isothermal–isobaric (NPT) ensemble at P = 1 atm and T = 300 K. The pressure and temperature were maintained with the Nosé–Hoover Langevin piston pressure control (oscillation period 200 fs, damping timescale 100 fs) [32,33] and the Langevin dynamics (damping coefficient = 5 ps^−1^) [34]. The cutoff distances were 12 Å for both electrostatic and van der Waals interactions, with switching to the smoothing function at 10 Å. The Particle-Mesh Ewald method was used to evaluate the long-ranged electrostatic interactions. A periodic boundary condition in 3 dimensions was also used. The integration step was set to 2 fs with the SHAKE/SETTLE algorithms [35,36] applied to constrain bonds to hydrogen atoms. The VMD 1.9.3 software [37] was used for the preparation of the model systems and the subsequent analysis of molecular dynamics (MD) trajectories.

The preparation of ORF2p complexes for the production runs consisted of two steps: the equilibration of water shells and the equilibration of the entire system. All simulations were performed as discussed above. The first step was performed in 2 ns molecular dynamics simulations, keeping the coordinates of the protein, DNA, RNA, dTTP frozen. The second step was performed in a 5 ns MD run with a harmonic constraint potential of 0.1 kcal/Å^2^ on the CA atoms of the protein and P atoms of DNA/RNA. Next, a 500 ns simulation was performed.

The molecular dynamic trajectories of all 4 complexes (ternary complex, DNA complex, RNA complex and apo-form of ORF2p) were prepared for analysis by the extraction of protein heavy atoms. TTClust 4.6 [38] and MDTraj 1.9.7 [39] software packages were used for the primary analysis of the protein RMSD and clustering of the obtained data. The EnGens workflow [24], which includes MDTraj 1.9.7 [39], PyEMMA 2.5.7 [40], and scikit-learn 1.5.1 [41], was employed for advanced feature extraction and clustering. The CA distance matrix from the calculated trajectories was employed as a feature matrix for further analysis with machine learning techniques. The residue CA picking stride was set to 25, thus yielding 51 CA atoms, which results in 1275 total features. The stride was chosen to limit the size of the feature matrix, as using all the CA atoms would have resulted in 812,175 features. The principal component analysis (PCA), with an 80% variance explained threshold, was used to reduce the number of dimensions in the studied space, followed by clustering in a reduced-dimensional space.

## 4. Conclusions

In this study, we conducted a series of sub-microsecond all-atom molecular dynamics simulations of the entire ORF2p complex with different starting configurations, which resulted in a total of over 61 thousand frames. The PCA dimensionality reduction technique, followed by the gaussian mixture models clustering, were employed for the feature matrix of CA–CA distances. Five and six clusters were obtained for the “open-ring” and “closed-ring” starting models, respectively. A close inspection of the representative structures of the obtained clusters and interdomain distance distributions within clusters yielded valuable insights into the dynamical behavior of the ORF2p complex, which is hard to uncover with experimental methods, given the complexity and size of the object.

The fingers–palm–thumb core retains its rigid configuration during the MD simulations, similarly to the experimental structures. All the other domains display the complicated dynamic behavior not observed in the experiment. While only the “thumb up” conformation is observed in all trajectories, the active site can be obstructed by the movement of the CTD domain. The EN and CTD domains display significant translations and rotations while their internal structures stay rigid. The CTD domain can either form strong contacts with the tower or be far apart from it for both formal “open-ring” and “closed-ring” states, as the tower hairpin position is not the only determining factor of the protein complex configuration. By redefining the criterion of “openness” and “closeness” through the minimal distance between the CTD and the tower domains to being less than 8–10 Å, we obtain 39–45% of “close contact” conformations for the trajectories started from the “open-ring” structure and 65–68% for the trajectories started from the “closed-ring” initial structure, respectively.

## Figures and Tables

**Table 1:** Backbone RMSD (in Å) calculated over MD frames, mean values and standard deviation (in parenthesis). MD frames were aligned over protein backbone atoms of the FPT core, either on the initial structure, or on the average structure. Here, and in the following text, all systems (1–4) assigned to the same type (“open” or “closed”), according to the initial state, were considered together during the data analysis. Time-dependent RMSD plots are shown in Figure S1.

	System Type	Open		Closed	
	Reference	Initial Structure	Average Structure	Initial Structure	Average Structure
**Domains**	**EN**	25.7(13.2)	21.5(8.1)	34.0(17.9)	21.4(10.8)
	**tower**	10.7(2.4)	9.4(2.0)	9.1(2.4)	6.9(1.7)
	**FPT core**	2.8(0.6)	2.2(0.5)	3.3(0.7)	2.3(0.5)
	**wrist**	8.7(2.5)	5.3(1.7)	8.3(2.2)	7.0(2.7)
	**CTD**	19.8(9.1)	17.7(8.4)	21.6(12.0)	16.0(9.0)

## Data Availability

The original contributions presented in the study are included in the article/Supplementary Materials, further inquiries can be directed to the corresponding author.

## Supplementary Materials

The supporting information can be downloaded at https://www.mdpi.com/article/10.3390/ijms26010073/s1.
