# Supplementary material for: Domain Mobility in the ORF2p Complex Revealed by Molecular Dynamics Simulations and Big Data Analysis
Source: Int J Mol Sci. 2024 Dec 25;26(1):73. doi: 10.3390/ijms26010073 (PMC11719894; doi:10.3390/ijms26010073)
Supplement: Supplementary file 1 [file ijms-26-00073-s001.zip › ijms-3342917-supplementary.pdf]

Supporting information for

Domain mobility in the ORF2p complex revealed by molecular dynamics simulations and big data analysis

Anna M. Kulakova[1,2], Maria G. Khrenova[1,3*], Maria I. Zvereva[1], Igor V. Polyakov [1]

[1] Chemistry Department, Lomonosov Moscow State University, 119991 Moscow, Russia

[2] Institute of Biomedical Chemistry, 119121, Moscow, Russia

[3] Bach Institute of Biochemistry, Federal Research Centre “Fundamentals of Biotechnology” of the Russian Academy of Sciences, 119071 Moscow, Russia

* Corresponding author: Prof. Maria G. Khrenova, E-mail: khrenovamg@my.msu.ru

S1. RMSD calculated over MD trajectories

Figure S1 demonstrates results of the MD simulations of 8 different model systems. Those are divided into two groups. The first group is composed of systems obtained from the “open-ring” structure (upper panel of Figure S1) and the second from the “closed-ring” structure (lower panel of Figure S1). Within these groups we considered four models: (1) ternary complex, (2) DNA complexes obtained from ternary complexes by removing dTTP nucleoside, (3) RNA complexes of ORF2p containing only RNA-template and protein and (4) apo-form of ORF2p. These systems were analyzed together within the same group, and the RMSD plots over time correspond to concatenated MD trajectories. All alignments were performed over the FPT core, reference structures were initial structures of the corresponding models. Large RMSD values and their fluctuations (Table 1) for the EN and CTD mean that these domains can be found in different places relative to the FPT core and corresponding domains in reference structures. The supporting materials deposited at ZENODO (see main text) allow one to visualize each MD trajectory and find that these domains themselves are rigid but mobile relative to the protein core.

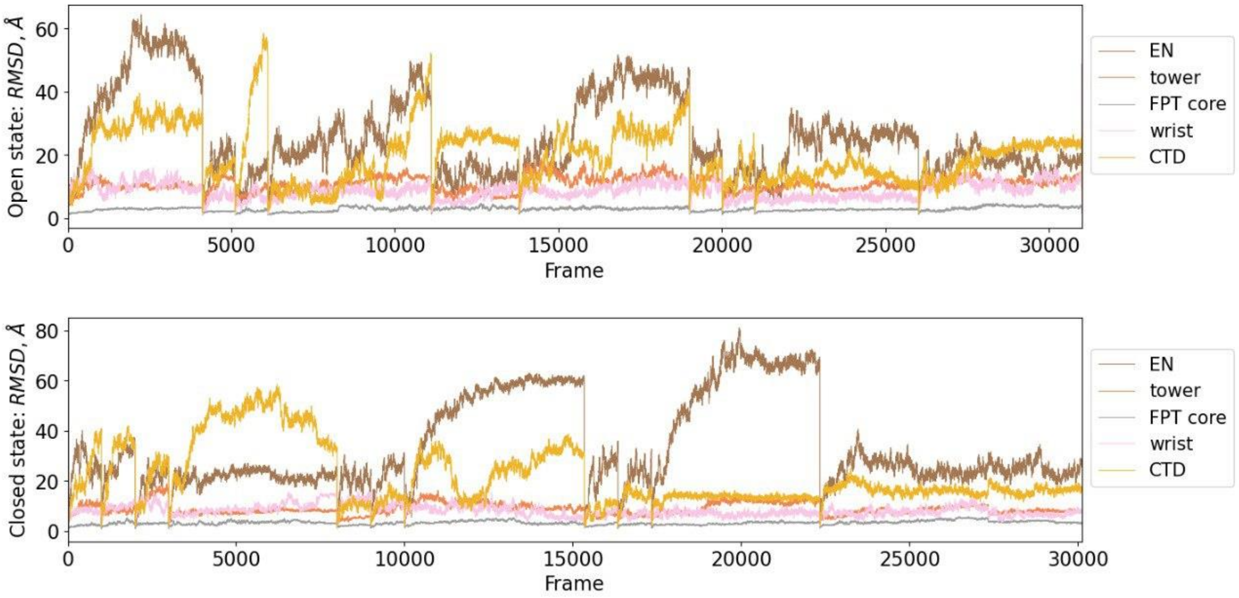

Figure S1. Time-dependent RMSD plots for “open-ring” and “closed-ring” systems. Alignment is performed over backbone atoms. Reference structures are initial protein structures from the corresponding groups, “open-ring” or “closed-ring” systems. The instantaneous jumps in the RMSD when switching between runs are observed as 17 and 16 runs were produced in total for the “closed-ring” and “open-ring” model systems respectively.

S2. Elbow analysis

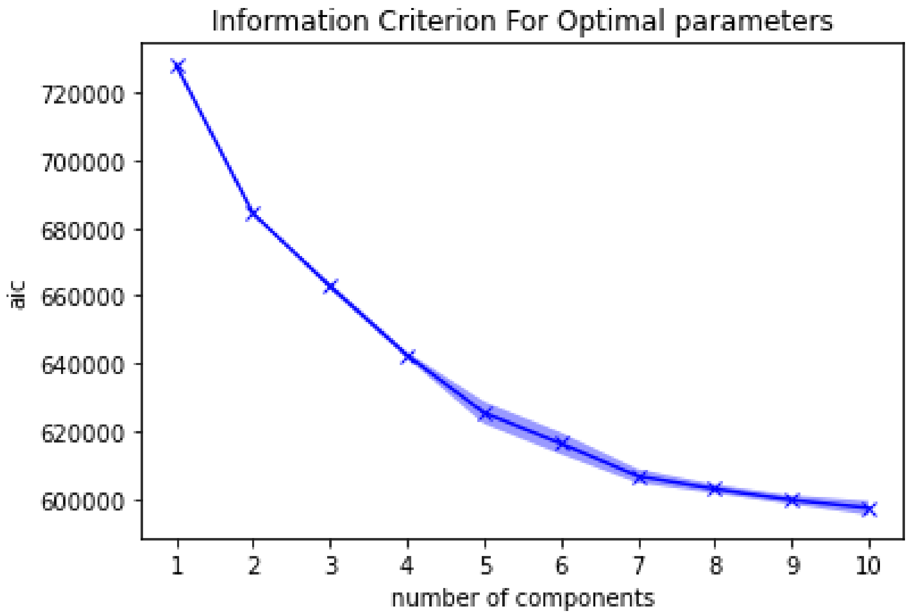

Figure S2. Elbow analysis for the “open-ring” model system.

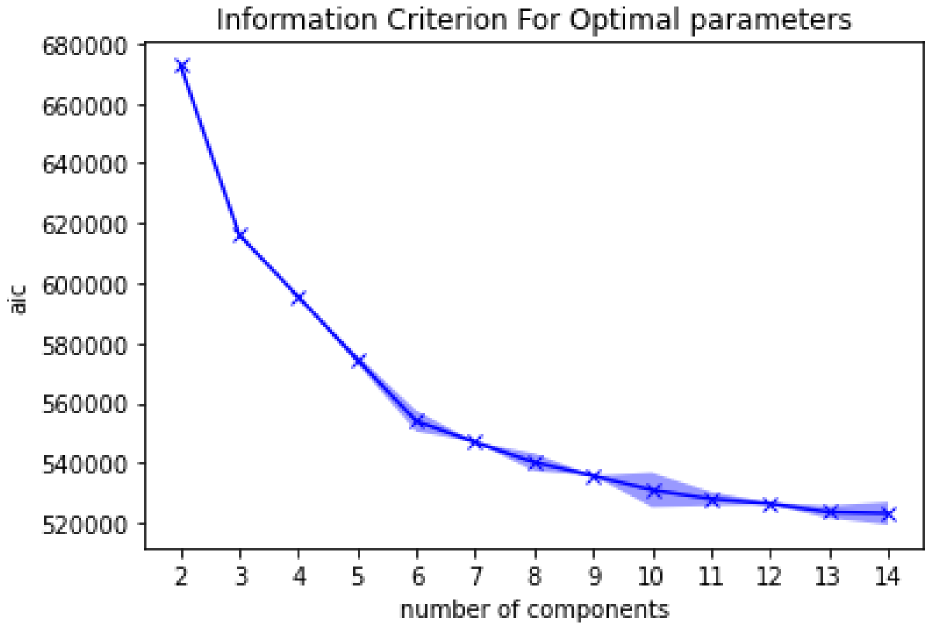

Figure S3. Elbow analysis for the “closed-ring” model system.

S3. Population of clusters in “open-ring” and “closed-ring” states

Table S1. Population of clusters in the “open-ring” models. For system notation see Models and Methods section.

System	Cluster population, %				
	#0	#1	#2	#3	#4
all	0	0	68.2	0	31.8
DNA	0	58.2	41.8	0	0
RNA	0	0	27.2	72.8	0
apo-form	58.9	0	10.7	30.4	0

Table S2. Population of clusters in the “closed-ring” models. For system notation see Models and Methods section.

System	Cluster population, %					
	#0	#1	#2	#3	#4	#5
all	3.5	0	29.1	67.4	0	0
DNA	31.3	0	0	4	64.6	0
RNA	99.8	0	0	0.2	0	0
apo-form	24.2	17.1	0	6.9	1.8	50
